# Recent Progress in Photodetectors: From Materials to Structures and Applications

**DOI:** 10.3390/mi15101249

**Published:** 2024-10-11

**Authors:** Tianjun Ma, Ning Xue, Abdul Muhammad, Gang Fang, Jinyao Yan, Rongkun Chen, Jianhai Sun, Xuguang Sun

**Affiliations:** 1 School of Electronics and Communication Engineering, Quanzhou University of Information Engineering, Quanzhou 362000, China; matianjun@qzuie.edu.cn (T.M.);; 2 State Key Laboratory of Transducer Technology Institute of Electronics, Chinese Academy of Sciences, Beijing 100190, China

**Keywords:** photodetectors, two-dimensional materials, quantum dots

## Abstract

Photodetectors are critical components in a wide range of applications, from imaging and sensing to communications and environmental monitoring. Recent advancements in material science have led to the development of emerging photodetecting materials, such as perovskites, polymers, novel two-dimensional materials, and quantum dots, which offer unique optoelectronic properties and high tunability. This review presents a comprehensive overview of the synthesis methodologies for these cutting-edge materials, highlighting their potential to enhance photodetection performance. Additionally, we explore the design and fabrication of photodetectors with novel structures and physics, emphasizing devices that achieve high figure-of-merit parameters, such as enhanced sensitivity, fast response times, and broad spectral detection. Finally, we discuss the demonstration of new applications enabled by these advanced photodetectors, including flexible and wearable devices, next-generation imaging systems, and environmental sensing technologies. Through this review, we aim to provide insights into the current trends and future directions in the field of photodetection, guiding further research and development in this rapidly evolving area.

## 1. Introduction

Photodetectors are vital components in modern technology, with applications ranging from environmental monitoring to communication systems, imaging, and medical diagnostics [1]. The ability to detect light and convert it into an electrical signal is a fundamental process that has driven the development of various devices, including cameras, solar cells, and optical sensors [2,3]. Over the past few decades, the field of photodetection has undergone significant transformation, propelled by advances in material science, device engineering, and the emergence of new applications demanding higher performance.

The discovery and development of emerging photodetecting materials have opened up new avenues for innovation. Traditional photodetectors, primarily based on silicon, face limitations in sensitivity, speed, and the range of detectable wavelengths [4,5,6,7,8,9,10]. In contrast, new materials such as perovskites [11,12], polymers [13,14,15], two-dimensional (2D) materials [16,17], and quantum dots (QDs) [18,19,20] offer unique properties that can overcome these limitations. For instance, perovskites are known for their exceptional light absorption and charge transport capabilities, while 2D materials like graphene and transition metal dichalcogenides (TMDs) exhibit remarkable electronic and optoelectronic properties, including high carrier mobility and tunable bandgaps [21,22,23,24,25]. These emerging materials not only enhance the performance of photodetectors but also enable the development of devices with novel functionalities.

The integration of these emerging materials into photodetectors has led to the creation of devices with novel structures and enhanced physical properties, resulting in substantial improvements in key performance metrics such as responsivity, detectivity, response speed, and spectral range [26,27,28,29,30]. For example, quantum dots offer size-tunable optical properties, allowing photodetectors to be designed for specific wavelength ranges, from ultraviolet (UV) to infrared (IR). Similarly, 2D materials like MoS_2_ and WS_2_ provide excellent opportunities for ultra-thin, flexible, and transparent photodetectors that can be seamlessly integrated into wearable devices and other flexible electronics. These advancements are not limited to enhancing performance but also include novel device architectures such as phototransistors, photonic crystals, and plasmonic-enhanced photodetectors, which leverage the unique properties of the materials to achieve highly efficient light matter interactions [31,32,33,34,35].

**Figure 1:**
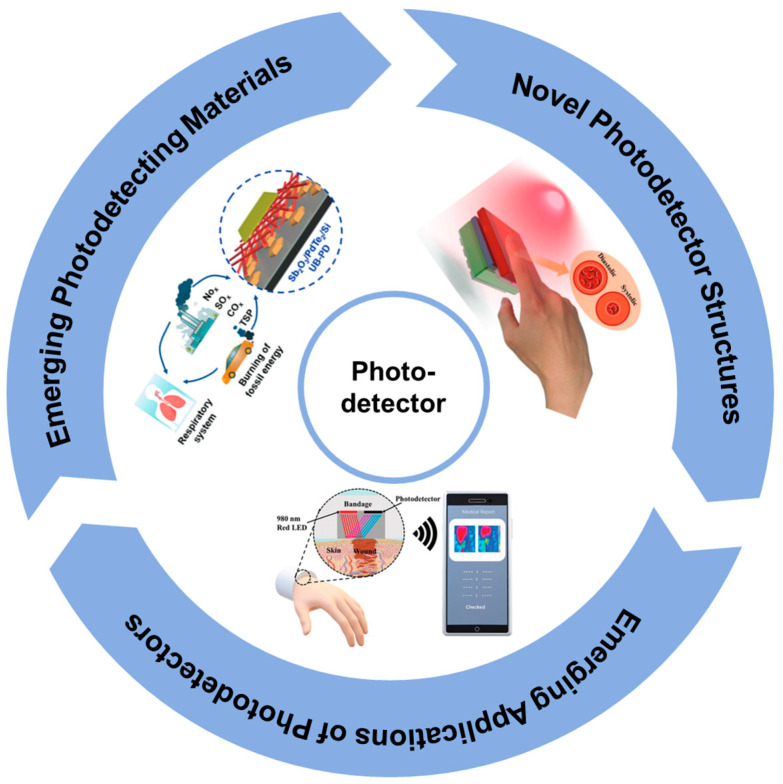
Design and application of photodetectors [36,37,38].

In parallel with material and structural innovations, new applications for photodetectors continue to emerge, particularly in fields such as healthcare and environmental sensing as shown in Figure 1. The ability to detect light at different wavelengths, combined with high sensitivity and fast response times, makes these advanced photodetectors ideal for use in non-invasive medical diagnostics, where precision and real-time monitoring are critical. In environmental monitoring, photodetectors can be employed in pollution detection and atmospheric studies, detecting subtle changes in light intensity due to particulates or gases.

This review will provide a comprehensive overview of recent advancements in photodetector technologies, focusing on three major areas: (1) the synthesis of emerging photodetecting materials, including perovskites, polymers, two-dimensional materials, and quantum dots; (2) novel photodetector structures and the impact of these materials on their performance; and (3) the demonstration of new applications that are driving the future of photodetector research. By examining these developments, we aim to highlight the potential of these technologies to reshape the landscape of photodetection and their integration into future optoelectronic systems.

## 2. Synthesis of Emerging Photodetecting Materials

The development of photodetectors has experienced transformative progress, largely driven by significant advancements in material science. These breakthroughs, particularly in the synthesis of novel materials, have led to a new generation of photodetecting devices with enhanced optical, electrical, and mechanical properties [39,40,41,42]. The continuous exploration of emerging materials, such as perovskites, organic polymers, two-dimensional (2D) materials, and quantum dots, has opened up avenues for creating high-performance photodetectors that not only surpass conventional technologies but also introduce entirely new capabilities. These materials exhibit unique properties that offer flexibility in design and functionality, allowing for the tailoring of photodetectors to meet the stringent requirements of various modern applications.

Perovskites, with their remarkable light absorption and tunable bandgaps, provide an efficient platform for capturing a wide range of wavelengths, from ultraviolet to infrared, making them ideal for applications in imaging, environmental monitoring, and telecommunications. Organic polymers, on the other hand, bring the advantage of mechanical flexibility, lightweight construction, and the ability to be processed using cost-effective methods such as printing or solution-based techniques. This enables their integration into flexible electronics and wearable devices, positioning them as key components for next-generation health monitoring systems, flexible displays, and smart textiles.

The rise of two-dimensional materials, including graphene and transition metal dichalcogenides (TMDs), has further expanded the scope of photodetection technologies. These atomically thin materials exhibit exceptional electrical conductivity, tunable optical properties, and strong light-matter interaction, making them highly suitable for ultrathin, transparent, and flexible photodetectors. The ability to integrate 2D materials into transparent and flexible substrates provides a new dimension to photodetector design, particularly in wearable electronics, heads-up displays, and soft robotics.

Quantum dots, with their size-tunable optical and electronic properties, offer unparalleled precision in wavelength selectivity, making them ideal for highly sensitive and efficient photodetectors. The unique quantum confinement effects in QDs allow for the fine-tuning of their optical response across the UV, visible, and IR spectra. This tunability makes them highly versatile for applications in advanced imaging systems, optical communication, medical diagnostics, and environmental sensing. Moreover, their compatibility with solution-processable techniques allows for scalable fabrication on large-area flexible substrates, broadening their application in transparent and flexible optoelectronics.

Beyond their individual advantages, the ability to control the synthesis and integration of these emerging materials plays a pivotal role in optimizing device performance [43,44,45,46,47,48]. Precise control over material composition, structure, and interface quality is critical in improving key photodetector performance metrics such as sensitivity, responsivity, noise equivalent power (NEP), and detectivity. Different material synthesis methods, including solution-based methods, chemical vapor deposition (CVD), spin coating, and mechanical exfoliation, exhibit significant differences in achieving material properties and performance, and their synthesis conditions have a direct impact on the final properties of the materials.

Solution-based methods are commonly used for material synthesis and are especially suitable for the preparation of nanomaterials and thin films. During synthesis, factors such as solution concentration, reaction temperature, and reaction time are crucial in determining material quality. By adjusting the solution concentration, one can control the crystallinity and particle size of the material, while controlling the reaction temperature and time can affect the uniformity and stability of the film. For example, by precisely controlling the temperature and time during the reaction, high-quality films can be obtained with smooth surfaces and uniform thickness, which are essential for effective light absorption and carrier mobility in optoelectronic applications.

Chemical vapor deposition (CVD) is an ideal choice for the synthesis of 2D materials. A significant advantage of the CVD method is its capability to produce uniform, layer-controllable films on a large scale. By adjusting reaction conditions, such as gas flow rate, deposition temperature, and reaction time, different layer numbers and crystal quality of 2D materials can be obtained. Specifically, for materials like MoS_2_, the CVD method can precisely control the thickness and crystal orientation, thus improving the optoelectronic properties of the film. In addition, spin coating is widely used in the preparation of solution-based materials to produce uniform films. During the spin-coating process, by changing the spin rate and duration, the thickness and uniformity of the film can be adjusted. Higher spin rates can reduce solvent residues, resulting in a uniform film. The post-spin thermal treatment also significantly influences the electrical properties of the material; proper annealing temperature and time can improve the crystallinity of the film, reduce interface defect density, and thus enhance device performance.

Mechanical exfoliation is suitable for preparing single-layer 2D materials under laboratory conditions. Although this method is not suitable for large-scale production, it is often used in research because it can yield high-purity, defect-free single-layer materials with optimal material properties. Quantum dot materials are typically synthesized through colloidal methods. By controlling the concentration of reactants, reaction temperature, and time, the size of quantum dots can be precisely controlled, thereby tuning their optical and electrical properties. Smaller quantum dot sizes result in blue-shifted absorption spectra, suitable for applications requiring high energy gaps, whereas larger quantum dots are more suitable for optoelectronic conversion applications with lower energy gaps. Furthermore, colloidal synthesis allows for adjustments in surface ligands, thereby improving the stability and charge transport efficiency of quantum dots. Advancements in the synthesis of these materials enable the development of novel device architectures, such as multi-junction or heterostructure-based photodetectors, which leverage the complementary properties of different materials to achieve superior performance across a broad spectral range.

In addition to traditional applications, these materials hold great promise for enabling entirely new classes of devices, particularly in the areas of flexible, lightweight, and transparent photodetectors. These devices are well suited for integration into wearable electronics, where their mechanical flexibility and lightweight nature are essential. For example, photodetectors integrated into wearable health monitoring systems can continuously track vital signs such as heart rate or blood oxygen levels, enabling real-time, non-invasive medical diagnostics. Similarly, transparent photodetectors could be integrated into displays or windows, offering new functionalities in consumer electronics and smart buildings.

### 2.1. Perovskite-Based Photodetectors

**Figure 2:**
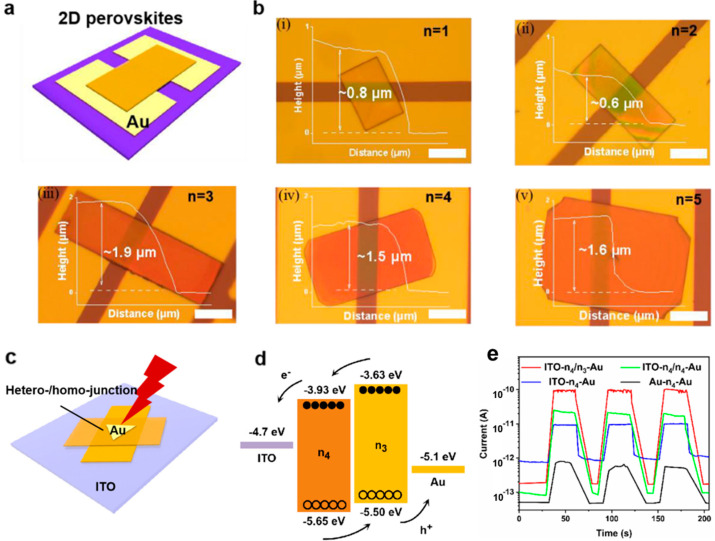
Microscope photographs of the device structure and the performance of 2D single-crystalline microplate photodetectors. (**a**) Schematic diagram of the device structure. (**b**) Photodetectors based on (BA)_2_(MA)_n−1_PbnI_3n+1_ microplate stacking on Au electrodes (images i–v correspond to *n* = 1–5, respectively). Scale bar: 15 μm. (**c**) Schematic diagram of hetero-/homostructure-based photodetectors. (**d**) Band alignment diagram of the (BA)_2_(MA)_3_Pb_4_I_13_/(BA)_2_(MA)_2_Pb_3_I_10_ heterostructure. (**e**) Self-powered property comparison of the ITO-n_4_/n_3_-Au, ITO-n_4_/n_4_-Au, ITO-n_4_-Au, and Au-n_4_-Au PDs. Semilogarithmic I-t curves under 600 nm illumination at 0 V [51].

Perovskites, particularly lead-halide perovskites (ABX_3_), have attracted considerable attention due to their exceptional optoelectronic properties, including high absorption coefficients, long carrier diffusion lengths, and tunable bandgaps [49,50]. Their synthesis methods, such as solution processing, vapor deposition, and spin coating, allow for low-cost and scalable production, making them ideal for large-area photodetector applications [9,18,51,52,53,54]. Recent research has focused on improving the stability of perovskites, which tend to degrade under environmental factors like moisture, heat, and light exposure [55]. By incorporating additives, passivation techniques, and improved encapsulation, researchers have enhanced the operational stability of perovskite photodetectors. Furthermore, the bandgap tunability of perovskites allows for the detection of a broad spectrum of light, from ultraviolet to near-infrared (NIR), making them versatile candidates for multi-spectral photodetectors. Hong et al. introduced a surface tension-dominant crystallization technique for developing single perovskite crystals, which were employed in vertically oriented hetero-/homojunction photodetectors, as illustrated in Figure 2 [51]. Halide perovskites, particularly of the Ruddlesden-Popper (RP) type, exhibit remarkable optoelectronic properties, including high absorption coefficients, tunable bandgaps, and strong quantum confinement effects. These attributes are key to enhancing the performance of photodetectors. The study utilized composition engineering to optimize the quantum well index, and it was observed that perovskites with a quantum well index of *n* = 4 delivered superior performance metrics. Specifically, these perovskite-based photodetectors exhibited an enhanced photocurrent, a diminished dark current, and an on/off ratio exceeding 3.5 orders of magnitude. Additionally, the study found that when operating in a self-powered mode, these photodetectors maintained high sensitivity and low power consumption, making them suitable for next-generation optoelectronic applications. The vertical junction structure played a crucial role in enhancing light absorption and charge separation, contributing to the overall efficiency of the device [51].

**Figure 3:**
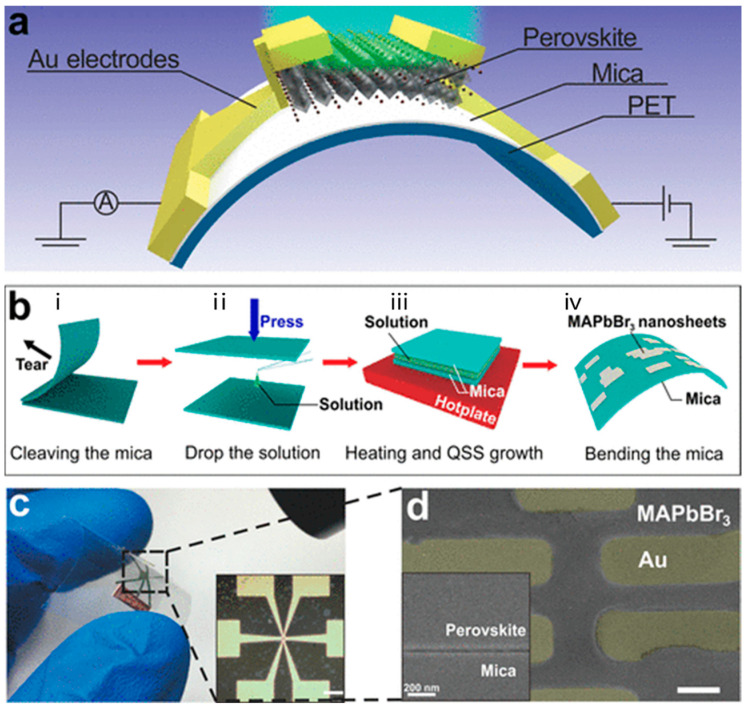
Architecture of the flexible photodetector and the characterization of the active layer. (**a**) Schematic of the device. (**b**) Schematic representations of the solution growth including the following four steps: (i) cleaving the mica, (ii) dropping the solution between the micas, (iii) heating and quasi-static solution (QSS) growth, and (iv) bending the mica substrate. (**c**) Photograph of the device. Inset: micrograph of the device, with a scale bar of 100 μm. (**d**) False-color SEM image of the device, where light yellow outlines the Au electrodes, and the scale bar is 3 μm. Inset: tilted SEM image of the edge of the perovskite nanosheet on mica [11].

**Figure 4:**
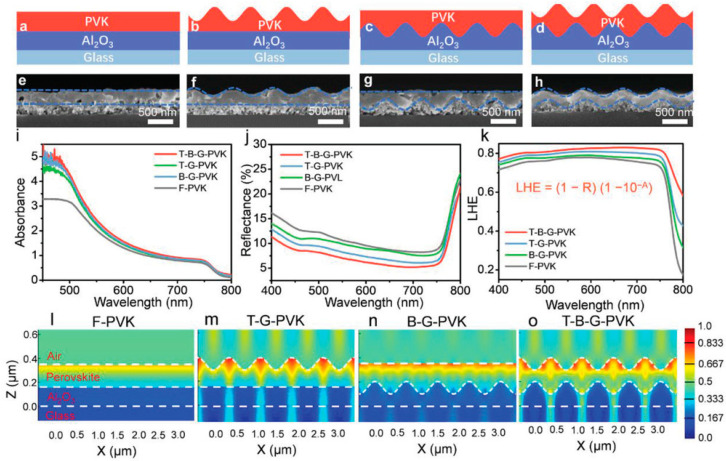
Characterization of light-trapping capability. (**a**–**d**) Schematic structure. (**e**–**h**) Cross-sectional SEM images, blue dashed lines marks the edge of PVK films. (**i**–**k**) Absorbance spectra, reflectance spectra, and light-harvesting efficiency. (**l**–**o**) Field plots of time-averaged electromagnetic energy density with respect to the *x*–*z* plane at the wavelength of 650 nm of F-PVK, T-G-PVK, B-G-PVK, and T-B-G-PVK films. T-B-G-PVK exhibits the highest light-harvesting capability [56].

Hong et al. developed a flexible ultrathin single-crystalline perovskite photodetector to enhance performance in wearable optoelectronic devices (Figure 3 shows the design) [11]. The researchers utilized CH_3_NH_3_PbBr_3_ perovskite, known for its superior photoelectric conversion efficiency compared to polycrystalline versions. By reducing the perovskite film to just 20 nm thickness, they significantly improved flexibility while maintaining excellent optical properties. The photodetector demonstrated a remarkable responsivity of 5600 A/W, outperforming other flexible perovskite devices by two orders of magnitude. It also exhibited fast response times, with a 3.2 μs rise time and a 9.2 μs fall time, making it ideal for high-speed photodetection. Furthermore, the device showed great mechanical durability, maintaining stable performance after thousands of bending cycles. This ultrathin design reduced photocurrent degradation often observed in thicker films, thereby enhancing overall performance. Song et al. developed a high-sensitivity perovskite photodetector utilizing a moiré pattern structure to enhance light absorption and polarization sensitivity, as shown in Figure 4 [56]. By incorporating a dual-grating structure, the authors created a photodetector that capitalizes on the moiré effect, significantly improving light-harvesting capabilities. The perovskite material CH_3_NH_3_PbX_3_ (X = Cl, Br, I) was selected due to its excellent optoelectronic properties, such as strong absorption and high carrier mobility. The results demonstrated a substantial increase in both responsivity (up to 15.62 A/W) and detectivity (up to 5.58 × 10^13^ Jones), outperforming traditional flat perovskite photodetectors. The unique design also enabled the device to achieve high polarization sensitivity, making it highly suitable for digital polarization imaging applications.

### 2.2. Two-Dimensional (2D) Materials

Two-dimensional materials, particularly transition metal dichalcogenides (TMDs) such as MoS_2_, WS_2_, and graphene, exhibit remarkable electronic and optical properties due to their atomically thin structures [57,58,59,60,61,62]. These materials offer high carrier mobility, strong light–matter interaction, and the ability to confine electrons in two dimensions, leading to enhanced photodetection capabilities [63,64]. The synthesis of 2D materials is typically achieved through techniques such as mechanical exfoliation, chemical vapor deposition, and molecular beam epitaxy (MBE). Each method offers different advantages in terms of material quality [65,66], scalability, and integration with existing technologies [67,68,69]. For example, CVD-grown MoS_2_ and WS_2_ photodetectors have demonstrated excellent responsivity and detectivity, particularly in the visible spectrum [70,71,72]. Furthermore, 2D materials can be stacked to form van der Waals heterostructures, enabling multi-functional photodetectors with tunable optoelectronic properties. This makes 2D materials highly adaptable for applications in ultrathin, flexible, and transparent photodetectors [73,74].

**Figure 5:**
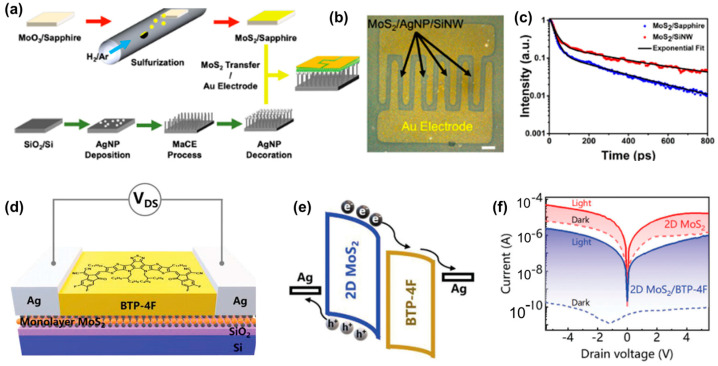
(**a**) Schematic illustration of the fabrication process of the bilayer MoS_2_, silicon nanowire, silver nanoparticle, and hybrid photodetector. (**b**) Optical image of the hybrid MoS_2_ device. Scale bar, 100 μm. (**c**) Normalized TRPL decay of bilayer MoS_2_ on SiNW and sapphire substrates. The exponential fits of both trends are shown with a black solid line [75]. (**d**) An illustration of the monolayer MoS_2_/BTP-4F device. (**e**) The energy-level marching for the monolayer MoS_2_ and BTP-4F film at the VDS of 5 V. (**f**) The I–V curves corresponding to the pure MoS_2_ and MoS_2_/BTP-4F devices, respectively [76].

Mao et al. developed a highly sensitive 2D photodetector based on a bilayer MoS_2_ combined with 1D silicon nanowires and 0D silver nanoparticles to enhance optoelectronic performance, as illustrated in Figure 5a–c [75]. The integration of silver nanoparticles leverages plasmonic effects to significantly improve light absorption and the interaction between light and the bilayer MoS_2_. The bilayer MoS_2_, known for its tunable band gap and high quantum yield, was chosen for its efficient charge carrier generation under light exposure. The results showed that the hybrid photodetector achieved a high responsivity of 402.4 A/W at 532 nm, outperforming other 2D photodetectors without a gate. Furthermore, the system demonstrated an excellent detectivity of 2.34 × 10^12^ Jones, making it ideal for advanced optoelectronic applications such as high-performance and low-power photodetectors. Xu et al. explored a highly efficient photodetector by combining 2D MoS_2_ with an organic material (BTP-4F) [76]. The integration of these materials results in a P-N heterojunction that facilitates ultrafast charge transfer and significantly reduces dark current. The 2D MoS_2_ offers excellent properties for photodetection, including high carrier mobility and strong light–matter interactions. Researchers formed a heterojunction with the organic material BTP-4F, which enhances the charge transfer efficiency and suppresses the dark current by four orders of magnitude, as illustrated in Figure 5d–f. The study demonstrated that the 2D MoS_2_/BTP-4F photodetector exhibits an ultrafast response time of 332/274 μs, a significant improvement over pure MoS_2_ devices, which have response times of around 1.2 s. This fast response is achieved due to the effective electron transfer between MoS_2_ and BTP-4F, which facilitates rapid separation of electron–hole pairs. The photodetector shows a high responsivity of 3.2 A W^−1^ and an external quantum efficiency (EQE) of 756%. The ultrafast charge transfer time of 0.24 ps was measured using time-resolved transient absorption spectroscopy, highlighting the photodetector’s potential for high-speed applications.

### 2.3. Quantum Dots in Photodetectors

Quantum dots are nanocrystals that exhibit size-dependent optical and electronic properties, making them ideal for wavelength-selective photodetectors. The tunable bandgap of QDs allows for precise control over the absorption spectrum, enabling detection across the UV, visible, and infrared ranges [18,19]. Synthesis methods for quantum dots, such as colloidal synthesis, provide a high degree of control over size, composition, and surface passivation, ensuring uniformity and stability in photodetector performance [77,78,79]. Quantum dot-based photodetectors can be incorporated into various device architectures, including phototransistors and photodiodes, where they offer high quantum efficiency, low noise, and fast response times [20]. Their compatibility with solution-based processing also allows for the fabrication of large-area devices on flexible or transparent substrates. Quantum dot photodetectors are particularly promising for applications requiring tunable detection capabilities [80], such as imaging, environmental sensing, and optical communications [81].

**Figure 6:**
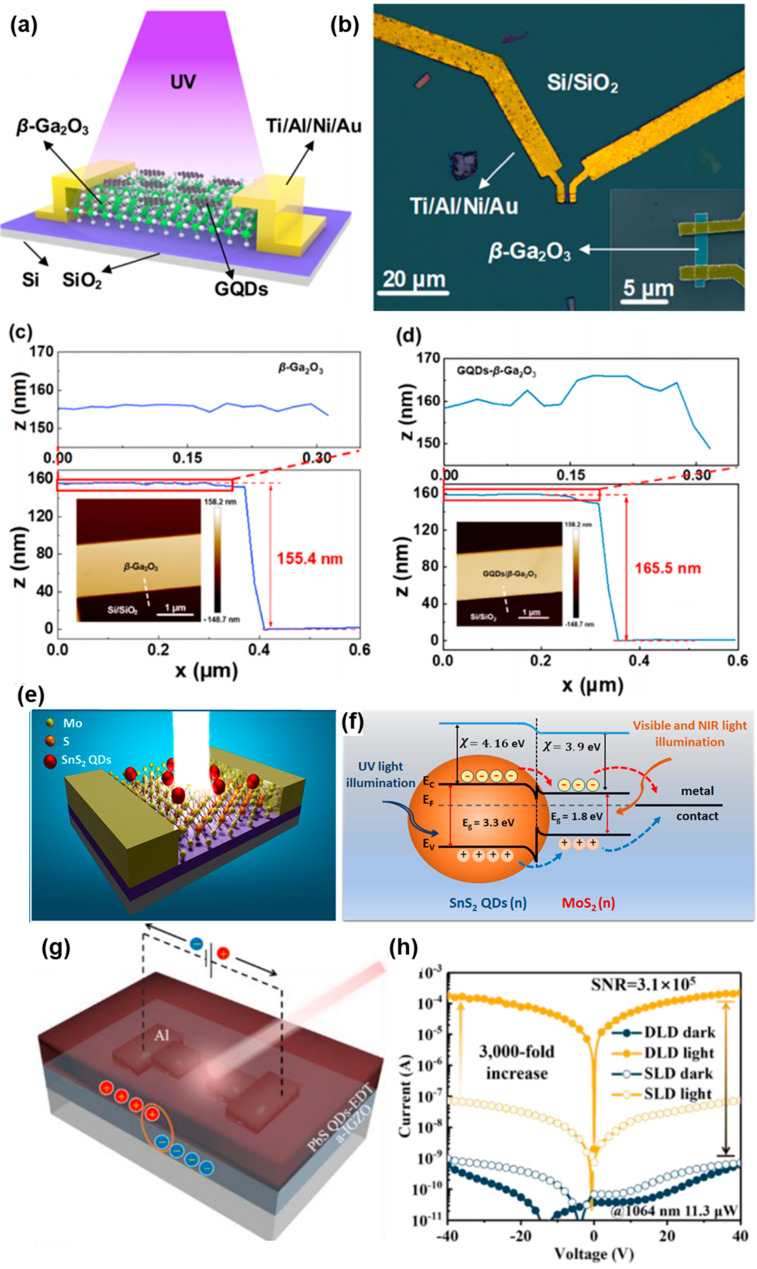
Fabrication and characterization of bare β-Ga_2_O_3_ and GQDs/β-Ga_2_O_3_ PDs. (**a**) Schematic diagram of the hybrid GQDs/β-Ga_2_O_3_ PD under light illumination. (**b**) Optical microscope image of the fabricated β-Ga_2_O_3_ device after annealing with the SEM image of the effective area of the β-Ga_2_O_3_ flake, as shown in the inset. (**c**) AFM images of the bare β-Ga_2_O_3_ device (left) and (**d**) the GQDs/β-Ga_2_O_3_ device (left) with a cross-sectional height profile (right) along the white dashed line depicted in the AFM images of (**c**,**d**). The enlarged images in (**c**,**d**) reveal that the size of these GQDs is ~10.1 nm [82]. (**e**) A 3D schematic representation of the MoS_2_/SnS_2_ QDs heterojunction. (**f**) Schematic illustration of the SnS_2_-QDs and monolayer MoS_2_ band structure after the formation of a heterojunction with proposed (**e**–**h**) pair separation [83]. (**g**) A 3D scheme of the a-IGZO/PbS QDs heterojunction device. (**h**) I–V curves under dark and NIR light (@1064 m, 11.3 μW) of the a-IGZO/PbS QDs heterojunction device and the PbS QDs-EDT film-only device [84].

As shown in Figure 6a–d, Zeng et al. present an advancement in photodetector technology by integrating graphene quantum dots (GQDs) with quasi-two-dimensional β-Ga_2_O_3_ [82]. This heterostructure exhibits enhanced photoresponsivity, shorter response times, and improved detection across a broad range of wavelengths, particularly in the deep-ultraviolet (DUV) region (200–280 nm). The hybrid device outperforms traditional β-Ga_2_O_3_ photodetectors by leveraging GQDs, which enhance light absorption and generate additional electron–hole pairs, leading to a superior responsivity of approximately 2.4 × 10^5^ A/W and a detectivity of 4.3 × 10^13^ Jones. The external quantum efficiency reaches 1.2 × 10^8^%, making this design promising for solar-blind photodetectors in applications such as missile tracking, UV sterilization, and environmental sensing. The integration of GQDs allows for broader spectral sensitivity, extending the detection range to the near-infrared region, which is a notable improvement over conventional β-Ga_2_O_3_ devices. The improved performance is attributed to efficient charge transfer facilitated by the quantum confinement effects of GQDs. Kolli et al. developed a broadband, ultra-high-responsive photodetector using a mixed-dimensional heterojunction of monolayer MoS_2_ and SnS_2_ quantum dots, as illustrated in Figure 6e,f [83]. The device combines the light absorption capabilities of SnS_2_ quantum dots with the excellent charge transport properties of 2D MoS_2_, creating a highly efficient photoactive interface. This hybrid structure enhances light–matter interactions and enables the photodetector to operate across a broad spectral range from ultraviolet to near-infrared. The results demonstrated that the hybrid photodetector achieved a responsivity of 278 A/W in the UV range, 435 A/W in the visible range, and 189 A/W in the NIR range. Additionally, the photodetector exhibited fast response times (~100 ms), making it suitable for applications requiring rapid detection and response. Zhang et al. developed a high-performance photodetector based on a heterojunction between amorphous indium gallium zinc oxide (a-IGZO) and lead sulfide (PbS) quantum dots (Figure 6g,h) [84]. The PbS quantum dots, known for their tunable bandgap and high optical absorption in the near-infrared (NIR) range, were combined with the a-IGZO layer, which acts as a photocurrent amplifier. This structure significantly enhances the photocurrent by up to 3000 times compared to standalone PbS quantum dot devices. The results demonstrated that the photodetector achieved outstanding performance, including a responsivity of 19,070 mA/W and a detectivity of 1.53 × 10^13^ Jones under NIR light.

## 3. Novel Photodetector Structures and Performance

The emergence of novel photodetector structures has significantly influenced the field, pushing the boundaries of device performance and enabling unique functionalities that were previously unattainable with conventional architectures. Innovations in device design, including heterostructures, multi-junction devices, plasmonic-enhanced photodetectors, and photonic crystal-based structures, have improved key performance metrics such as sensitivity, speed, and spectral range [85]. These new structures have been engineered to take advantage of the distinct optical and electronic properties of emerging materials, such as perovskites, two-dimensional materials, and quantum dots, further enhancing device efficiency and expanding their range of applications [86].

One of the key advancements in novel photodetector structures is the development of heterostructure-based designs, where multiple layers of different materials are stacked to create unique energy band alignments [87]. The specific stacking configuration and the interface quality between these layers play a crucial role in the efficiency of charge separation and transport, directly impacting the device’s overall responsivity and noise characteristics. Heterojunctions formed through these carefully engineered structures allow for more efficient charge separation and transport, which not only enhance the device’s responsivity but also significantly reduce noise.

Recent advances in heterostructure-based photodetectors include the integration of materials such as perovskites, transition metal dichalcogenides, and quantum dots into novel device architectures [86]. For instance, perovskite-based heterostructures have shown impressive performance improvements, particularly due to their high absorption coefficients and long carrier diffusion lengths. These characteristics have made them suitable for heterojunction designs, where efficient charge extraction is facilitated by the carefully tailored energy band alignment at the interfaces. Moreover, van der Waals heterostructures incorporating TMDs like MoS_2_ and graphene have demonstrated remarkable sensitivity due to their atomically sharp interfaces, which minimize defect-induced recombination and provide an excellent pathway for charge carrier transport. In particular, 2D materials like MoS_2_ and graphene have been effectively combined with conventional semiconductors or other 2D materials to form van der Waals heterostructures. The atomic-level precision in the arrangement of these layers enables highly sensitive detection due to their strong light–matter interactions and fast carrier mobility, which are highly dependent on the interlayer coupling and alignment. Additionally, new device configurations such as staggered type-II band alignment heterostructures have been utilized to achieve superior charge separation, leading to enhanced photodetection performance across a wide spectral range [88,89].

Furthermore, the tunability of band gaps in heterostructures is directly influenced by the number of layers and the way they are stacked, allowing for photodetectors with tailored spectral responses, from ultraviolet to infrared, which is crucial for applications in multi-spectral imaging and optical communication systems. Recent reports also highlight the use of quantum dot heterostructures, which combine the size-tunable optical properties of quantum dots with the high mobility of 2D materials to achieve broad spectral coverage and enhanced signal-to-noise ratios. These heterostructures can be designed to have strong light absorption and fast response times, making them highly effective for next-generation optoelectronic applications.

Multi-junction photodetectors represent another leap forward in device performance, with the structural arrangement being key to their capabilities. By stacking multiple absorbing layers, each optimized for a different portion of the electromagnetic spectrum, the structural design of multi-junction photodetectors allows them to capture a broader range of wavelengths compared to single-junction devices [90,91]. The alignment and thickness of these individual layers critically determine the absorption efficiency and the charge carrier collection, leading to a more comprehensive spectral response. This structural enhancement is particularly beneficial in applications such as solar-blind UV photodetection, infrared imaging, and environmental sensing, where detecting signals across different wavelength bands is essential. The combination of emerging materials such as perovskites or quantum dots with multi-junction architectures, carefully designed to optimize layer stacking and interfacial properties, has led to significant improvements in detectivity and noise-equivalent power, making these devices ideal for high-performance, broadband photodetection.

Plasmonic-enhanced photodetectors represent a novel approach to boosting the efficiency of light absorption and improving the sensitivity of photodetectors, where the physical placement and integration of plasmonic elements are critical to their functionality. By integrating metal nanostructures into the photodetector architecture, surface plasmon resonances are induced, which concentrate light into sub-wavelength volumes, thereby increasing the local electromagnetic field intensity. The position, size, and shape of these plasmonic nanostructures play a significant role in the extent of the enhancement. This structural enhancement leads to more efficient photon absorption, even in ultrathin absorbing layers, improving the overall quantum efficiency of the device. Plasmonic photodetectors are particularly useful in applications that demand high sensitivity, such as low-light imaging, optical sensors, and high-speed communications. The integration of plasmonic structures with materials like graphene, 2D TMDs, or quantum dots can further boost their performance, with the interfacial engineering and spatial arrangement of these elements being key factors in determining device efficacy. This makes such devices suitable for advanced applications in fields like biosensing and wearable technology, where structural precision and material interactions are paramount.

Another significant innovation is the use of photonic crystal-based structures in photodetector design. Photonic crystals are periodic optical nanostructures that can control the propagation of light, allowing for the design of devices with tailored optical properties. By incorporating photonic crystals into the photodetector architecture, it is possible to engineer devices with highly selective wavelength responses, narrowband filters, or enhanced light-trapping capabilities. These properties are particularly useful in applications such as spectral imaging, where detecting specific wavelengths with high precision is crucial. Additionally, photonic crystals can be combined with 2D materials or quantum dots to create highly sensitive, wavelength-specific photodetectors with minimal cross-talk and enhanced performance.

In addition to the advancements in device architecture, the integration of these novel structures with advanced fabrication techniques has enabled the development of flexible and transparent photodetectors. The use of flexible substrates, combined with emerging materials and novel device architectures, allows for the production of photodetectors that can be bent, stretched, or twisted without significant performance degradation. This opens the door to applications in wearable electronics, where photodetectors can be seamlessly integrated into clothing or skin-like devices for continuous health monitoring. Transparent photodetectors, on the other hand, offer new possibilities for integration into displays, windows, or transparent surfaces, providing additional functionalities such as biometric sensing or gesture recognition without compromising aesthetic or functional design.

### 3.1. Flexible and Transparent Photodetectors

Flexible and transparent photodetectors have garnered significant attention in recent years due to their potential for integration into next-generation wearable electronics, biomedical devices, and transparent displays. These photodetectors, which combine mechanical flexibility with optical transparency, enable novel applications such as skin-like sensors for continuous health monitoring, transparent security systems, and interactive displays embedded in glass surfaces [92]. The unique material properties of emerging photodetecting materials, including organic polymers, perovskites, and two-dimensional materials like graphene and MoS_2_, have been key enablers for these innovations [86].

The development of flexible photodetectors requires substrates that can withstand repeated mechanical deformation without compromising device performance. Polymeric substrates like polyethylene terephthalate (PET), polyethylene naphthalate (PEN), and polydimethylsiloxane (PDMS) are commonly used due to their mechanical robustness and optical transparency. Combined with thin, flexible layers of emerging materials, these substrates allow for photodetectors that can be bent, stretched, or twisted, maintaining high performance even under extreme mechanical strain. This flexibility opens up applications in wearable technology, where sensors embedded in clothing or directly applied to the skin must conform to dynamic human motion [93]. For instance, flexible photodetectors integrated into health-monitoring devices can continuously track vital signs like heart rate and oxygen saturation in real time, providing critical data without restricting the wearer’s movement [94,95].

Transparent photodetectors, on the other hand, are designed to be integrated into windows, displays, or other transparent surfaces without disrupting the visual aesthetics of the device. Transparent conducting electrodes, such as indium tin oxide (ITO) or graphene, are essential components of these photodetectors, allowing for the transmission of light while maintaining electrical conductivity [96,97]. By using transparent materials such as graphene or certain organic semiconductors, these photodetectors can be seamlessly integrated into smart windows that can detect light intensity, adjust transparency, or serve as touch-sensitive panels. Additionally, in augmented reality systems, transparent photodetectors can be used to track gestures or environmental changes, enhancing user interaction without the need for external sensors. Two-dimensional materials, particularly graphene and transition metal dichalcogenides like MoS_2_, have also played a crucial role in the advancement of flexible and transparent photodetectors. These atomically thin materials exhibit excellent electrical conductivity, high carrier mobility, and strong light–matter interactions, enabling ultrafast response times and high sensitivity. Additionally, their mechanical properties make them inherently flexible, allowing for the fabrication of ultrathin, bendable photodetectors that can be integrated into a variety of surfaces. For instance, graphene-based transparent photodetectors can be incorporated into AR glasses, allowing for real-time environmental sensing without obstructing the user’s vision.

**Figure 7:**
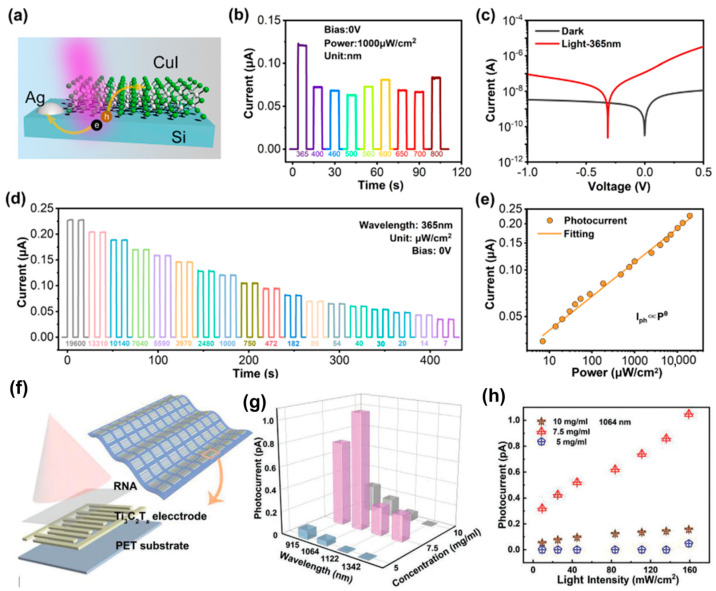
(**a**) Schematic diagram of the CuI/Si photodiode. (**b**) Time-resolved photoresponse of the device at 0 V bias under illumination with different monochromatic light wavelengths. (**c**) Current–voltage characteristics of the CuI/Si photodetector in the dark and under illumination with 365 nm light at 1000 μWcm^−2^. (**d**) Transient responses of the CuI/Si photodetector under various light intensities of 365 nm light at 0 V bias. (**e**) Light–power-dependent photocurrent of the photodetector under 365 nm light irradiation at 0 V bias [98]. (**f**) Schematic diagram of a single Ti_3_C_2_T_x_-RAN PD structure. (**g**) Photocurrent curves of devices with concentrations of 10, 7.5, and 5 mg mL^−1^ at 915, 1064, 1122, and 1342 nm. (**h**) Plots of photocurrent changes with different optical power densities for devices with concentrations of 10, 7.5, and 5 mg mL^−1^ at a wavelength of 1064 nm [99].

**Figure 8:**
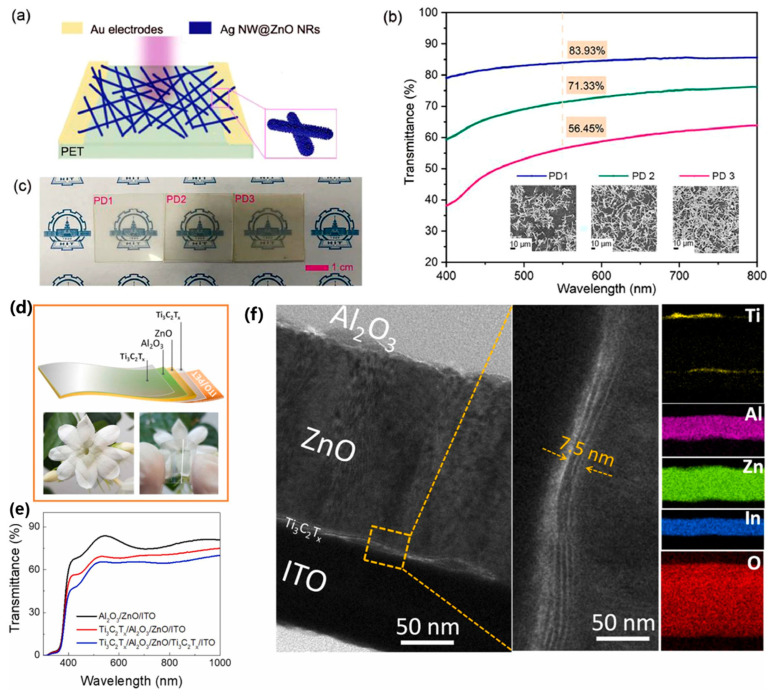
(**a**) Schematic illustration of the Ag NW@S-ZnO NR-based UV detector. (**b**) UV–Vis optical transmittance spectra of Ag NW@S-ZnO NR thin films with different drop-coating times. The insets are corresponding SEM images of the Ag NW@S-ZnO NRs network of PD1, PD2, and PD3. (**c**) Appearance of Ag NW@ZnO NR film on HIT-logo paper [100]. (**d**) Schematic diagram of the Ti_3_C_2_T_x_/Al_2_O_3_/ZnO/Ti_3_C_2_T_x_/ITO/PET device, accompanied by digital images of it. (**e**) Transmittance profiles of the ZnO-based flexible photodetector with and without Ti_3_C_2_T_x_. (**f**) Cross-section TEM image of the Ti_3_C_2_T_x_/Al_2_O_3_/ZnO/Ti_3_C_2_T_x_/ITO/PET device, along with elemental mapping profiles of Ti, Al, Zn, In, and O [101].

The combination of flexibility, transparency, and high performance in photodetectors represents a significant advancement in the field of optoelectronics. These devices are poised to play a critical role in the development of wearable technology, smart windows, and transparent electronic systems, offering new possibilities for seamless integration into everyday life. For example, the copper iodide (CuI) films exhibit high transparency, with an average transmittance of 91% in the visible light spectrum (400–800 nm), making them ideal for transparent applications. Huang et al. developed a transparent, flexible ultraviolet (UV) photodetector utilizing p-type CuI thin films for enhanced performance in wearable electronics, as shown in Figure 7a–e [98]. By fabricating the photodetector on a flexible polyimide substrate, the device maintained its transparency and functionality even after repeated bending, demonstrating significant mechanical durability. The photodetector achieved a high responsivity of 123.3 mA/W and a detectivity of 5.7 × 10^12^ Jones, with fast response times (rise time of 90 ms and decay time of 140 ms). The device’s performance remained stable under mechanical stress, making it highly suitable for applications in transparent displays, wearable technology, and environmental monitoring systems. Hu et al. developed a flexible and transparent near-infrared photodetector based on a Ti_3_C_2_Tx MXene-RAN van der Waals heterostructure, as shown in Figure 7f–h [99]. The photodetector efficiently detects NIR light by leveraging the unique properties of the MXene material, which offers excellent conductivity and transparency, and the organic RAN layer, which serves as the photosensitive material. The device achieved a 6.25-fold improvement in the on–off ratio compared to conventional gold electrode-based photodetectors under NIR light (1064 nm). The photodetector exhibited high transparency (>70%) in the visible spectrum and maintained its performance after repeated bending, highlighting its flexibility and durability. The heterostructure allows for enhanced charge transfer, improving the overall responsivity and signal-to-noise ratio. Zhang et al. developed a flexible and transparent ultraviolet photodetector based on a cross-linked Ag nanowire @ZnO nanorod (NR) structure to enhance UV light absorption and mechanical flexibility, as shown in Figure 8a–c [100]. The Ag NW@ZnO NRs hybrid system offers a high surface-to-volume ratio, which significantly improves the photodetector’s sensitivity to UV light, particularly at 365 nm. The photodetector is fabricated on a flexible PET substrate, allowing it to maintain high transparency with an average transmittance of over 70% in the visible light spectrum. The device maintains stable performance even after 1000 bending cycles at a 120° angle, showcasing its mechanical durability and potential for use in wearable electronics and flexible displays. This innovative approach addresses traditional limitations in ZnO nanorod-based photodetectors, offering a scalable and efficient solution for high-performance, flexible UV photodetection. Nguyen et al. developed a flexible and transparent MXene-based ultrafast photodetector for encrypted signal communication and self-powered operation, as illustrated in Figure 8d–f [101]. The photodetector is built on a polyethylene terephthalate (PET) substrate, providing flexibility and transparency, which are essential for wearable electronics and optical communication applications. The device operates in a self-powered mode, utilizing a built-in pyro-phototronic effect, eliminating the need for an external power source. The photodetector achieves an ultrafast photo-response of 8 µs and a responsivity of 0.34 A/W, making it suitable for high-speed optical communication tasks, such as processing encrypted optical signals like Morse code. The Ti_3_C_2_T_x_ MXene layer acts as an efficient charge transport layer, enhancing the performance of the photodetector by improving charge separation and transport.

### 3.2. Photonic Crystal and Nanostructured Photodetectors

Photonic crystal (PhC) and nanostructured photodetectors have opened up exciting new avenues in photodetection technology by leveraging the unique optical properties of nanoscale architectures. Photonic crystals are periodic optical structures that can manipulate the flow of light by creating photonic bandgaps, much like how semiconductor crystals create electronic bandgaps [102]. By incorporating photonic crystals or other nanostructured materials into photodetector designs, it is possible to achieve unprecedented control over light–matter interactions, resulting in enhanced sensitivity, wavelength selectivity, and reduced noise [103,104].

The primary advantage of photonic crystal-based photodetectors lies in their ability to engineer the light propagation pathways through the device. Photonic bandgap effects allow for precise filtering of specific wavelengths, enabling narrowband detection without the need for external optical filters. This capability is particularly beneficial for applications requiring high spectral resolution, such as hyperspectral imaging, environmental monitoring, and biological sensing [105,106]. Additionally, the ability to confine light within the photonic crystal structure can significantly enhance the absorption efficiency of photodetectors, even in thin active layers. This enhanced absorption is critical for low-light or high-speed applications where maximizing photon capture is essential for performance.

**Figure 9:**
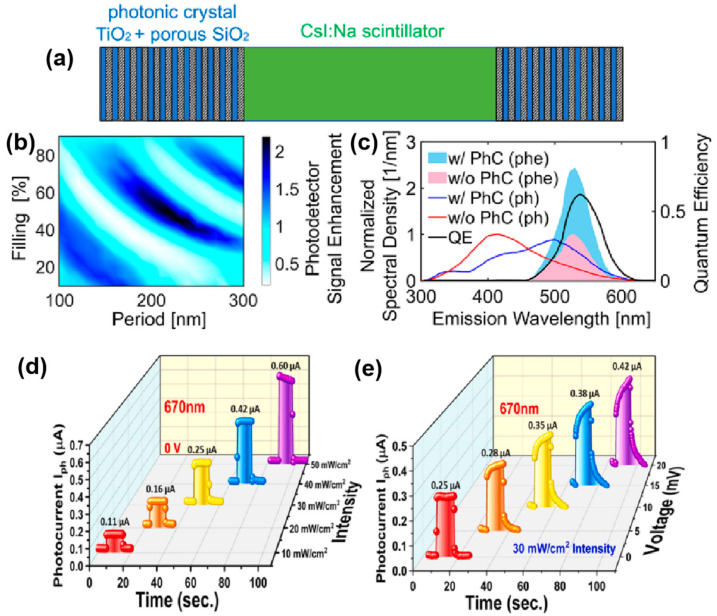
(**a**) Schematic diagram of the CsI:Na scintillator with a PhC cavity (TiO_2_ + porous SiO_2_). The TiO_2_/SiO_2_ PhCs have been shown to be feasible in previous work. Here, we replace the SiO_2_ film with a porous SiO_2_ material so as to achieve a low refractive index (1.06) for the SiO_2_ layer. The porous SiO_2_ material can be realized by random SiO_2_ nanorod arrangement. (**b**) Photodetector signal enhancement vs. period and filling factor of the photonic crystal. (**c**) Spectral density of photons (ph) and photoelectrons (phe) with optimized configuration of the PhC cavity (TiO_2_ + porous SiO_2_). The emission spectrum is shifted to a longer wavelength to match the quantum efficiency spectrum of the photodetector [107]. (**d**) The pulse photoresponse of the photodetector for red light with varying intensity at non-biased and self-biased conditions. (**e**) Different bias voltages at a 30 mW/cm^2^ intensity [108].

Ye et al. developed a photonic crystal (PhC) cavity system to enhance the detection capabilities of large-area scintillators, as shown in Figure 9a–c [107]. The PhC cavities are integrated externally with scintillators to enhance light extraction efficiency via the Purcell effect, which significantly improves the overlap between the scintillator’s emission spectrum and the photodetector’s quantum efficiency. This results in an enhanced photodetector signal, particularly in applications such as high-energy particle detection and medical imaging. The results show that the introduction of PhC cavities can boost the photodetector signal by over 200%, without requiring modifications to the scintillator material itself. The external addition of PhC structures allows for flexible design and improves the performance of large-area photodetectors, such as those used in positron emission tomography and security-scanning systems. This approach highlights the potential of photonic crystal-based enhancements for photodetectors, offering a scalable and efficient way to improve detection sensitivity in various large-scale imaging applications. As shown in Figure 9d,e, Jagani et al. developed a photonic crystal-based photodetector to enhance light absorption and detection efficiency [108]. The device utilizes a tin mono-selenide photonic crystal structure, which was fabricated using a direct vapor transport technique. The high-quality SnSe crystal allows the photodetector to operate effectively under various light conditions, including both monochromatic (red and blue) and polychromatic light sources. The results indicate that the photonic crystal structure significantly improves the photodetector’s sensitivity and responsivity, especially under blue light, due to the higher photon energy. Moreover, the device can function under self-biased conditions, making it ideal for low-power applications where external power sources are limited. The study showcases the potential of using photonic crystal structures in photodetectors to improve performance across different wavelengths, highlighting the benefits of SnSe-based materials for optoelectronic applications, including environmental sensing and imaging.

In addition, the electrode structure plays a critical role in photodetectors, directly affecting the device’s light absorption efficiency, carrier collection efficiency, and overall responsivity. Firstly, the shape and material selection of the electrodes has a direct impact on light incidence and the optical absorption characteristics of the device. Transparent electrodes, such as ITO or graphene, are often used as the top electrode to maximize light incidence and reduce reflection losses. In addition, the thickness and surface morphology of metallic electrodes also influence the optical path of the photodetector. For instance, using a nanomesh or ultra-thin metal electrode design can effectively reduce light reflection, thereby improving the light absorption efficiency of the device [80].

Secondly, the position and arrangement of the electrodes are crucial for the effective collection of carriers. In traditional planar electrode structures, carriers must travel through the entire active layer to be collected, which can lead to higher recombination losses. In contrast, in three-dimensional or grid electrode structures, the interface between the electrodes and the active layer is closer to the carrier generation sites, which can significantly reduce the carrier transport path, thereby decreasing recombination losses and improving response speed. Moreover, the choice of electrode material is essential for determining the contact characteristics of the photodetector, directly impacting carrier injection and collection efficiency. For instance, selecting an electrode material with a work function that matches the active material can form ohmic contacts, reducing contact resistance and enhancing effective carrier collection. Inappropriate electrode materials, however, may lead to Schottky barriers, increasing the difficulty of carrier collection and ultimately affecting the overall device performance.

## 4. Emerging Applications of Photodetectors

As advancements in photodetector technology continue to accelerate, the development of novel devices with enhanced capabilities is opening up a broad spectrum of applications across multiple industries. These cutting-edge photodetectors are not only becoming more sensitive but also achieving faster response times and greater precision in controlling light–matter interactions. These improvements are driving innovation in areas that were previously limited by slower, less accurate, or less responsive photodetection technologies [5,7,109]. This rapid progress is leading to the emergence of new applications and use cases, many of which are significantly reshaping the landscape of various fields while also creating opportunities for the development of entirely new categories of devices [110,111].

In consumer electronics, photodetectors are enabling more advanced features in devices such as smartphones, cameras, and wearable technology. Higher sensitivity and faster response times allow for better low-light performance in cameras, leading to improved image and video quality, even in challenging lighting conditions. Additionally, photodetectors are crucial in facial recognition technology, where their precision helps enhance security and user authentication. Wearable devices, like smartwatches and fitness trackers, utilize advanced photodetectors for health monitoring, such as measuring heart rate and blood oxygen levels, and even tracking sleep patterns with greater accuracy.

In healthcare, the role of photodetectors is becoming increasingly vital. They are integral to non-invasive medical imaging techniques, such as optical coherence tomography (OCT), which provides detailed images of biological tissues, particularly in ophthalmology. Photodetectors also support innovations in biosensing, enabling the detection of specific biomarkers in bodily fluids for early disease diagnosis. These advances allow for more accurate monitoring of patient health and facilitate personalized medicine by enabling continuous and real-time data collection. Environmental monitoring is another field seeing significant benefits from advanced photodetector technologies. For instance, highly sensitive photodetectors can be used to measure pollutants in the air and water with unprecedented accuracy, helping to track environmental changes and pollution levels in real time. These real-time data are crucial for early detection of environmental hazards, such as wildfires or toxic gas leaks, enabling quicker response times and minimizing potential damage. Additionally, photodetectors play a key role in monitoring changes in atmospheric conditions, contributing to more accurate climate models and weather predictions.

Beyond these sectors, photodetectors are also driving innovation in the automotive industry, particularly in autonomous vehicles. LIDAR (Light Detection and Ranging) technology, which relies on photodetectors, enables vehicles to map their surroundings with high precision, improving navigation and obstacle detection in real time. This technology is essential for the development of safe and efficient self-driving cars. Moreover, photodetectors are increasingly being used in advanced driver-assistance systems (ADAS), improving safety by helping to detect pedestrians, road signs, and other vehicles, even in low-visibility conditions.

In the field of communications, photodetectors are key components in optical fiber networks, where they convert light signals into electrical signals to transmit vast amounts of data at high speeds. The improved sensitivity and speed of modern photodetectors are enhancing the performance of optical communication systems, contributing to the growth of 5G networks and beyond. These advancements support the increasing demand for faster and more reliable data transmission, which is crucial for the expansion of the Internet of Things (IoT), smart cities, and other data-intensive applications.

Emerging applications in quantum computing and cryptography are also leveraging the capabilities of advanced photodetectors. In quantum computing, precise photodetectors are essential for detecting single photons and enabling quantum state measurements, which are critical for the development of quantum processors. Similarly, in quantum cryptography, photodetectors are used in secure communication systems to detect quantum keys, providing a higher level of security than classical cryptographic methods.

As the performance of photodetectors continues to improve, their potential applications are expanding into even more novel and futuristic domains. From space exploration, where photodetectors can aid in capturing distant celestial phenomena, to next-generation displays and augmented reality (AR) systems, photodetectors are at the forefront of technological innovation. Their ability to manipulate and sense light with increasing precision opens the door to new possibilities across a diverse range of industries.

### 4.1. Healthcare and Biometric Sensors

Photodetectors are playing an increasingly pivotal role in advancing healthcare and biometric technologies due to their ability to provide non-invasive, real-time monitoring with high precision. These sensors are at the core of medical devices used to track physiological parameters, offering a range of applications that enhance both diagnosis and treatment in modern medicine. Their integration into wearable technologies has been transformative, enabling continuous monitoring outside of clinical settings, thereby reducing the need for frequent hospital visits and allowing early detection of potential health issues [112].

One of the most widely recognized uses of photodetectors in healthcare is in pulse oximetry, where the sensors measure oxygen saturation in the blood. Pulse oximeters use LEDs to emit light at two wavelengths, typically red and infrared. Photodetectors then measure the amount of light absorbed by the blood, which varies according to oxygen levels. This technology provides critical real-time data in various settings, including hospitals, home care, and during surgery. Heart rate monitors and blood pressure-monitoring systems utilize photodetectors to track blood flow changes. These devices rely on the photoplethysmography (PPG) technique, where photodetectors measure the volumetric changes in blood circulation as light is absorbed or reflected by tissues. Advances in flexible and transparent photodetectors have further expanded their applications in wearable healthcare devices, making it possible for patients to continuously monitor vital signs in real time, improving personal health management and reducing the burden on healthcare systems [38,113]. Another cutting-edge application involves continuous glucose monitoring (CGM), which traditionally relies on invasive techniques. Photodetectors are now being integrated into non-invasive glucose monitoring systems that measure glucose levels by detecting changes in skin optical properties or by using near-infrared (NIR) spectroscopy. These sensors offer significant promise for patients with diabetes, enabling frequent glucose monitoring without the need for needle pricks, thus improving patient compliance and health outcomes.

Beyond vital sign monitoring, photodetectors are also used in early detection of cardiovascular diseases. By analyzing changes in the vascular system through optical signals, photodetectors can help identify abnormalities in blood flow that might indicate underlying cardiovascular conditions, such as hypertension or arteriosclerosis. These innovations are particularly valuable in wearable devices that continuously assess cardiovascular health, offering a non-invasive and continuous method for early detection.

In addition to their extensive use in healthcare, photodetectors are also integral to biometric authentication systems, offering secure and highly accurate identification methods. Fingerprint recognition, one of the most common biometric applications, utilizes photodetectors to capture detailed images of the unique ridge patterns of an individual’s fingerprint. These systems use light-emitting diodes (LEDs) to illuminate the finger, and the photodetectors measure the light reflected back from the finger’s surface to create a precise fingerprint map. The introduction of infrared (IR) photodetectors in fingerprint scanners has greatly improved performance, allowing for more detailed imaging and higher resistance to external interferences such as dirt or oil on the skin.

**Figure 10:**
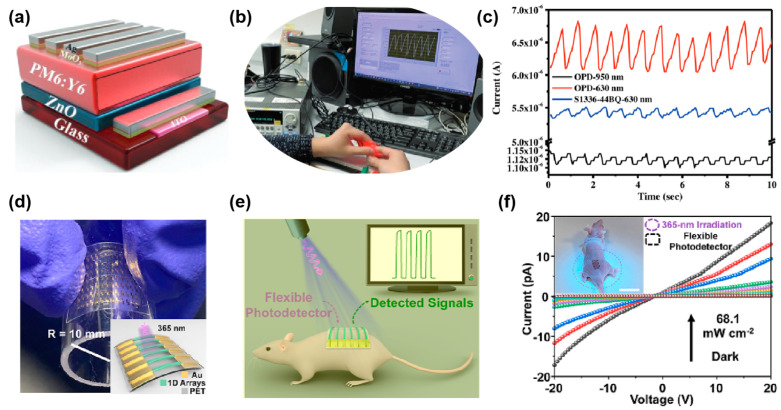
(**a**) Chemical structures of PM6 and Y6. (**b**) Finger photoplethysmography setup. (**c**) Direct current read-out of the PPG device using 950 and 630 nm LEDs [36]. (**d**) Photograph of a typical flexible photodetector based on 1D arrays bent at a bending radius of 10 mm, with the inset presenting the schematic illustration of the device. (**e**) Scheme of the flexible photodetectors monitoring the UV photodetection signals. (**f**) Typical I-V curves of the polymer array-based photodetectors under the dark condition and under different UV light illuminations, obtained from the attached flexible photodetector device on the back skin of the mouse. Inset: photograph of the flexible device attached closely to the back skin of a nude mouse [114].

As shown in Figure 10a–c, Yu et al. developed ambient-stable near-infrared organic photodetectors (OPDs) with ultrahigh detectivity and ultrafast response, primarily targeting applications in biometric monitoring, such as real-time heart rate measurement [36]. The flexible and lightweight OPDs were designed using organic photovoltaic materials, offering ease of integration into portable and wearable devices. The results demonstrated the potential of these OPDs for healthcare applications by achieving a high detectivity of 3.1 × 10^13^ Jones and a fast response time of 220 ns under ambient conditions, which makes them ideal for continuous biometric monitoring. These devices can monitor subtle changes in light absorption caused by blood flow, such as in photoplethysmography (PPG) for heart-rate monitoring. Zhang et al. developed ultraviolet photodetectors based on polymer microwire arrays designed for use in wearable medical devices, as illustrated in Figure 10d–f [114]. These flexible photodetectors are specifically tailored to monitor UV exposure on the skin in real time, making them ideal for continuous health-monitoring applications. The device leverages the high mechanical flexibility of the polymer microwire arrays, which allows it to maintain 97% of its original performance even after 4000 bending cycles, demonstrating its durability for wearable applications. These devices can be easily integrated into wearable technologies, making them a promising solution for next-generation health-monitoring systems, ensuring both comfort and reliability in medical applications.

The accuracy of these biometric systems largely depends on the sensitivity and resolution of the photodetectors used, as well as their ability to function in various lighting conditions. With advancements in OPDs and flexible materials, there is growing potential for invisible biometric sensors that can be seamlessly integrated into everyday objects like smartwatches, clothing, or even glasses, allowing for more natural and discreet forms of authentication. The development of flexible and transparent photodetectors has further broadened the scope of their applications, particularly in wearable devices and biometric authentication systems. These new photodetectors, made from materials like graphene, 2D materials such as MoS_2_, and quantum dots, offer enhanced mechanical flexibility while maintaining high performance in detecting light across various wavelengths. For instance, transparent photodetectors can be integrated into the display of a smartphone or smartwatch, allowing for continuous health monitoring without disrupting the aesthetic or functionality of the device. Moreover, flexible photodetectors can be embedded in smart fabrics or patches, offering a more seamless and comfortable solution for long-term health monitoring. These devices could track parameters like skin temperature, hydration levels, and muscle activity, and even function as early warning systems for conditions like heatstroke or dehydration.

### 4.2. Environmental and Atmospheric Sensing

Photodetectors are critical for environmental and atmospheric monitoring, where they are used to detect pollutants, monitor air quality, and measure changes in weather patterns. These sensors are employed in various environmental applications due to their high sensitivity to light and ability to detect minute changes in atmospheric conditions. For instance, UV photodetectors are integral to monitoring ozone depletion by measuring UV radiation levels in the atmosphere. The depletion of the ozone layer increases the amount of UV radiation reaching the Earth’s surface, which can be detrimental to ecosystems and human health. By continuously tracking UV levels, photodetectors help researchers assess the extent of ozone-layer damage and monitor the success of recovery efforts over time [115,116,117].

In addition, photodetectors are widely used in LIDAR (Light Detection and Ranging) systems for air-quality assessments. These systems utilize photodetectors to measure the backscattered light from airborne particles, such as dust, smoke, and pollutants, providing real-time data on particulate matter levels in the air. This information is crucial for cities and governments aiming to control air pollution and mitigate its harmful effects on public health. Photodetectors in LIDAR systems are also essential for studying aerosol compositions, cloud cover, and the dynamics of airborne particles during pollution events such as wildfires or dust storms.

In climate research, photodetectors play an essential role in tracking radiation balances between the Earth and its atmosphere. These sensors help monitor the albedo effect (the reflectivity of the Earth’s surface) and its impact on climate change. By analyzing the light reflected from clouds, snow, oceans, and vegetation, scientists can better understand how changes in surface characteristics affect the global climate system. Additionally, photodetectors are employed to monitor greenhouse gas concentrations (such as CO_2_ and methane), providing insights into their distribution and accumulation in the atmosphere, which is vital for tracking the progress of climate change [118,119].

**Figure 11:**
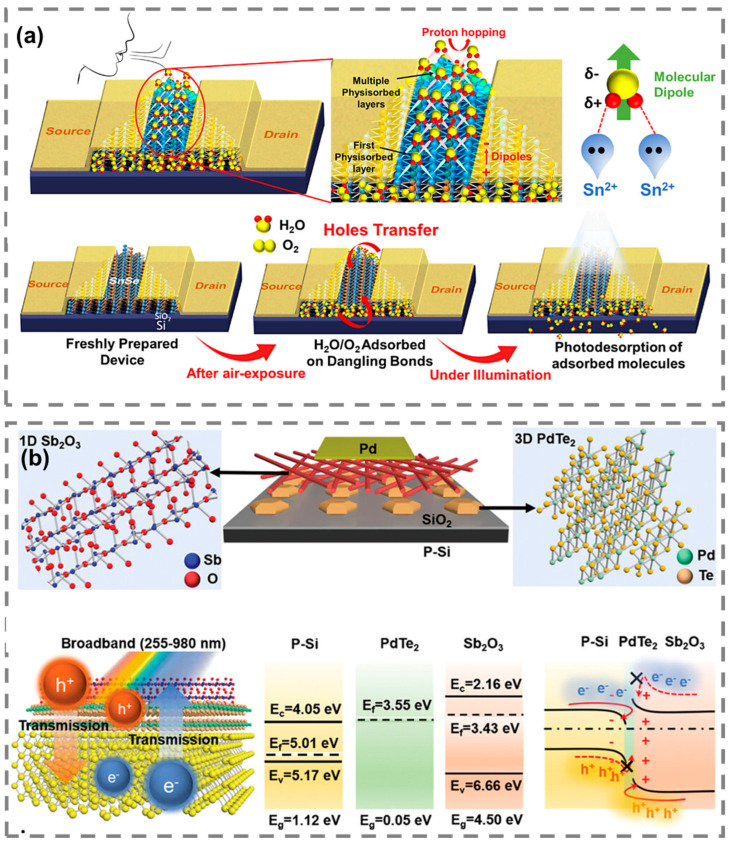
(**a**) The schematic representation of the device under humid conditions shows the formation of physisorbed layers and dipoles on the basal planes of SnSe, and a schematic representation of a freshly prepared pristine Au/Ti/SnSe/Ti/Au device after air exposure and under white light illumination [120]. (**b**) Structural schematic of a Sb_2_O_3_/PdTe_2_/Si heterojunction photodetector and a self-powered photoresponse mechanism of Sb_2_O_3_/PdTe_2_/Si heterojunction photodetectors [37].

Recent advancements in flexible and transparent photodetectors have significantly expanded their use in remote and harsh environments. Traditional rigid sensors are often unsuitable for deployment in extreme conditions, such as arctic regions, deserts, or areas prone to natural disasters. However, flexible photodetectors made from advanced materials like graphene and organic photodetectors offer greater adaptability and durability. These sensors can be integrated into wearable environmental monitors, providing individuals and field researchers with real-time data on air quality, UV exposure, and temperature. Such monitors are especially useful for those working in remote locations or in occupations with high exposure to environmental hazards, such as construction, mining, or forestry. Rani et al. developed an SnSe-based metal–semiconductor–metal (MSM) photodetector for environmental and atmospheric sensing applications [120]. As shown in Figure 11a, the device leverages the environmental sensitivity of SnSe to detect changes in humidity and other atmospheric conditions. The unique feature of this photodetector is its ability to transition from positive to negative photoconductivity (NPC) when exposed to ambient environmental factors like oxygen and water molecules. This environmental sensitivity allows the photodetector to be used as a highly responsive humidity sensor. The fast response and recovery times of the SnSe photodetector make it ideal for continuous environmental monitoring in various conditions. Wang et al. developed a self-powered broadband photodetector based on a mixed-dimensional Sb_2_O_3_/PdTe_2_/Si heterojunction, designed to detect a broad spectrum of light, as shown in Figure 11b [37]. The photodetector demonstrates high sensitivity across a wide wavelength range, from solar-blind ultraviolet (SBUV) to near-infrared (NIR), making it suitable for environmental and atmospheric monitoring applications. The photodetector is also capable of detecting environmental pollutants, such as NO_2_ and PM2.5, with high sensitivity, enabling real-time air-quality assessments. By integrating broadband detection with self-powered operation, this photodetector is highly efficient and versatile for environmental monitoring systems.

Moreover, flexible and lightweight photodetectors can be mounted on drones and autonomous vehicles, allowing for large-scale environmental surveillance and data collection over wide areas. Drones equipped with photodetectors can monitor atmospheric conditions in real time, capturing critical information on pollution hotspots, tracking weather changes, and measuring the impact of natural disasters such as hurricanes or volcanic eruptions. This enables more accurate environmental monitoring and predictive modeling, leading to better decision-making in disaster management, resource allocation, and environmental protection efforts [121,122].

### 4.3. Information Processing

Photodetectors play a crucial role in optical systems that require the precise detection and manipulation of light for encoding, transmitting, and processing data. Photodetectors are increasingly being integrated into optical communication networks, where they convert optical signals back into electrical signals, enabling faster and more efficient data transmission [112,123]. The high speed and bandwidth of light-based systems make photodetectors essential for meeting the growing demand for high-capacity data networks, especially with the increasing reliance on cloud computing, 5G networks, and the Internet of Things (IoT).

One of the key advancements in this field is the development of high-speed photodetectors capable of handling the increasing data rates in modern communication networks [124]. Traditional communication systems are being pushed to their limits as data traffic continues to grow exponentially. Photodetectors with greater responsivity, higher bandwidth, and lower noise are required to meet the demands of 5G technology, data centers, and edge computing. These advancements in photodetection enable faster data transmission rates, reduced latency, and more efficient energy consumption, which are critical for enabling high-speed data processing and communication [125,126,127].

**Figure 12:**
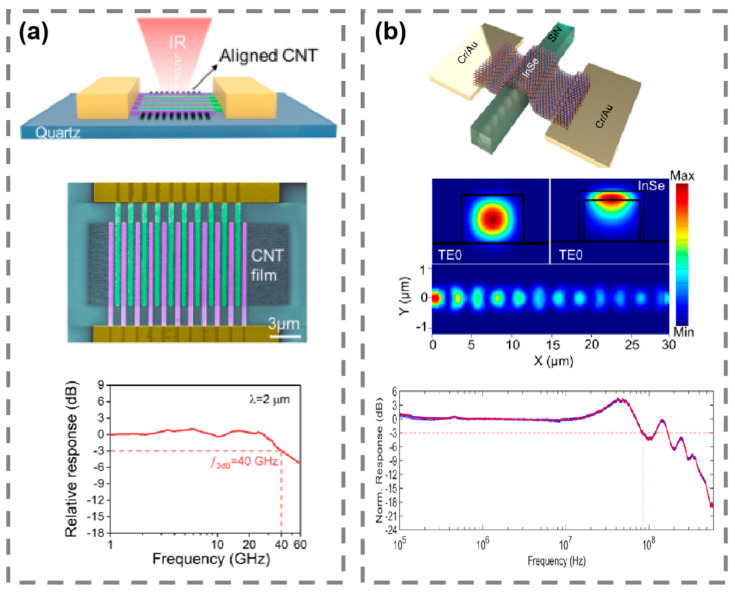
(**a**) Schematic diagram of the Pd-Hf-contacted CNT photodetector under illumination and a false-colored SEM image showing the channel of an as-fabricated CNT photodetector with Lch = 200 nm and total Wch = 200 μm. Relative response as a function of the modulation frequency of the input signal for the CNT photodetector. The extracted 3 dB bandwidth is 40 GHz at V = −0.2 V [131]. (**b**) A 3D cross-section representation of the heterogeneous InSe/SiN photodetector and electric-field profiles (|E|^2^) of TE modes of an unloaded SiN waveguide and 90 nm InSe on SiN at 976 nm (top panel). Normalized frequency response at 10 V. A total of 50 measurements (blue-scattered points) and average (red line) data are plotted. A 3 dB cut-off frequency of 85 MHz is measured [130].

Photodetectors are also critical in optical interconnects within data centers, where they facilitate communication between processors, memory modules, and storage systems. As the size of data centers grows, the need for efficient and scalable interconnect technologies becomes more pressing. Optical interconnects, which rely on photodetectors to convert optical signals into electrical signals, offer a significant advantage over traditional copper-based interconnects due to their higher bandwidth and lower energy consumption [128,129,130]. By integrating photonic technologies into these interconnect systems, data centers can handle more data with greater energy efficiency, thus supporting the ever-increasing computational demands of modern applications such as artificial intelligence, machine learning, and big data analytics. Wu et al. developed a high-speed carbon nanotube (CNT) photodetector for the 2 μm communication band, as shown in Figure 12a [131]. The device leverages the unique electrical properties of CNTs to achieve efficient photodetection, with a responsivity of 0.62 A/W and a 3 dB bandwidth of 40 GHz. The CNT photodetector outperforms conventional photodetectors in bandwidth, particularly in the 1.55 μm and 2 μm communication bands, which are critical for next-generation optical communication systems. These advancements make CNT photodetectors ideal candidates for high-speed data transmission and on-chip sensing in optical communication systems, as well as in imaging and sensing applications requiring rapid response times and high bandwidth. As illustrated in Figure 12b, Tamalampudi et al. developed a high-speed waveguide-integrated InSe photodetector on a silicon nitride (SiN) platform for NIR communication applications [130]. The device leverages the strong light absorption of InSe and the low-loss properties of the SiN waveguide to efficiently convert optical signals into electrical signals at high speeds. The photodetector achieves a photoresponsivity of 0.38 A/W and operates with a 3 dB bandwidth of 85 MHz, supporting data rates of up to 1 Gbps. The device exhibits a low dark current and high signal-to-noise ratios, making it suitable for high-speed optical communication and data processing. By integrating InSe into the SiN platform, the photodetector demonstrates enhanced dynamic response and compatibility with existing photonic circuits, offering significant potential for use in LiDAR, optical interconnects, and on-chip optical systems.

## 5. Conclusions

In summary, photodetectors based on perovskites, two-dimensional materials, and quantum dots have demonstrated exceptional optoelectronic properties, offering promising avenues for high sensitivity, flexibility, and tunable performance across various wavelengths. These materials provide the foundation for developing next-generation photodetectors with enhanced efficiency and stability. In terms of structural innovations, the development of flexible and transparent photodetectors, along with photonic crystal and nanostructured designs, has enabled devices that are more adaptable to diverse environments and applications. The ability to integrate photodetectors into wearable devices, transparent displays, and compact photonic systems demonstrates the versatility of these novel structures, improving both the performance and the usability of photodetectors.

The expanding range of applications, particularly in healthcare and biometric systems, environmental and atmospheric monitoring, and information processing, underscores the growing importance of photodetectors in modern technologies. These devices are now central to real-time monitoring, secure communication, and data-processing systems, offering improved speed, sensitivity, and adaptability in critical areas such as medical diagnostics, air-quality assessment, and high-speed optical communication.

As the field of photodetector research continues to evolve, future developments will likely focus on enhancing the performance, scalability, and integration of these devices into multifunctional systems. The progress outlined in this review suggests that photodetectors will remain at the forefront of technological innovation, driving advances in various industries and shaping the future of optoelectronics.

## Data Availability

Data are available upon reasonable request from the corresponding author.
